# Myricetin attenuates hypoxic-ischemic brain damage in neonatal rats *via* NRF2 signaling pathway

**DOI:** 10.3389/fphar.2023.1134464

**Published:** 2023-03-08

**Authors:** Tingting Chen, Yingying Hu, Liying Lu, Qianlei Zhao, Xiaoyue Tao, Bingqing Ding, Shangqin Chen, Jianghu Zhu, Xiaoling Guo, Zhenlang Lin

**Affiliations:** ^1^ Department of Pediatrics, The Second School of Medicine, The Second Affiliated Hospital and Yuying Children’s Hospital of Wenzhou Medical University, Wenzhou, Zhejiang, China; ^2^ Key Laboratory of Perinatal Medicine of Wenzhou, The Second Affiliated Hospital and Yuying Children’s Hospital of Wenzhou Medical University, Wenzhou, Zhejiang, China; ^3^ Basic Medical Research Center, The Second Affiliated Hospital and Yuying Children’s Hospital of Wenzhou Medical University, Wenzhou, Zhejiang, China; ^4^ Key Laboratory of Structural Malformations in Children of Zhejiang Province, The Second Affiliated Hospital and Yuying Children’s Hospital of Wenzhou Medical University, Wenzhou, Zhejiang, China; ^5^ Key Laboratory of Children Genitourinary Diseases of Wenzhou, The Second Affiliated Hospital and Yuying Children’s Hospital of Wenzhou Medical University, Wenzhou, Zhejiang, China

**Keywords:** neonatal hypoxic-ischemic (HI) brain injury, myricetin, oxidative stress, apoptosis, NRF2 signaling pathway

## Abstract

**Introduction:** Hypoxic-ischemic encephalopathy (HIE) is a crucial cause of neonatal death and neurological sequelae, but currently there is no effective therapy drug for HIE. Both oxidative stress and apoptosis play critical roles in the pathological development of HIE. Myricetin, a naturally extracted flavonol compound, exerts remarkable effects against oxidative stress, apoptosis, and inflammation. However, the role and underlying molecular mechanism of myricetin on HIE remain unclear.

**Methods:** In this study, we established the neonatal rats hypoxic-ischemic (HI) brain damage model *in vivo* and CoCl_2_ induced PC1_2_ cell model *in vitro* to explore the neuroprotective effects of myricetin on HI injury, and illuminate the potential mechanism.

**Results:** Our results showed that myricetin intervention could significantly reduce brain infarction volume, glia activation, apoptosis, and oxidative stress marker levels through activating NRF2 (Nuclear factor-E2-related factor 2) and increase the expressions of NRF2 downstream proteins NQO-1 and HO-1. In addition, the NRF2 inhibitor ML385 could significantly reverse the effects of myricetin.

**Conclusion:** This study found that myricetin might alleviate oxidative stress and apoptosis through NRF2 signaling pathway to exert the protective role for HI injury, which suggested that myricetin might be a promising therapeutic agent for HIE.

## 1 Introduction

Perinatal asphyxia-induced hypoxic-ischemic encephalopathy (HIE) is one of the important causes of neonatal death and nervous system dysfunction (Sun et al., 2017). The nearly half of HIE patients die in the neonatal period, and surviving infants have a high risk of neurological sequelae, which can cause global public health burden (Rocha-Ferreira and Hristova, 2015). HIE is not a single occurrence, but is an ongoing process that leads to neuronal death within hours to days after the initial injury (Jaworska et al., 2017). Despite the application of hypothermia as the reliable standard therapy for neonatal HIE, the treatment time window of hypothermia is very narrow (Sahuquillo and Vilalta, 2007; Goswami et al., 2020). The treatment effects of hypothermia on moderate or severe patients are very limited, and even have many adverse reactions (Michniewicz et al., 2022). Thus, it is imperative to explore more effective treatment strategies to improve the prognosis of neonatal hypoxic-ischemic (HI) brain damage.

With in-depth study, researchers found that the pathophysiology of neonatal HIE is a complex evolutionary process. In the initial stage of primary injury, the brain undergoes a series of energy-failure-related processes, such as excitotoxin accumulation, oxidative stress, and inflammation activation (Rodriguez et al., 2020), and the immature neonatal brain is particularly sensitive to these processes (Blomgren and Hagberg, 2006). After several hours, the brain will enter the second stage of injury, during which the cell apoptosis is induced by inflammation and oxidative stress (Krystofova et al., 2018). Meanwhile, the cell apoptosis is a key factor influencing the extent of HI injury and determining the size or shape of the central nervous system (Thornton et al., 2017). Therefore, the target for relieving oxidative stress and neuronal apoptosis may be an effective strategy for HIE treatment.

Nuclear factor-E2-related factor 2 (NRF2) is a major regulator against oxidative stress (Zenkov et al., 2017). Under the normally physiological conditions, NRF2 can bind to cytosolic Kelch-like ECH-associated protein 1 (KEAP1), and then be degraded by ubiquitination (Done and Traustadottir, 2016). When cells are stimulated by oxidative stress, NRF2 can dissociate from KEAP1 protein and perform nuclear translocation, which subsequently can enhance the transcriptional expressions of targets NAD(P)H quinone oxidoreductase-1 (NQO-1) and heme oxygenase-1 (HO-1) (He and Ma, 2012; Chatterjee and Bohmann, 2018). Simultaneously, with the stimulation of oxidative stress, cell apoptosis can also be activated. NRF2 is widely expressed in several tissues, and its role in the nervous system is becoming clear. Study in Alzheimer’s disease model had shown that NRF2 could ameliorate cognitive deficits and reduce Aβ deposition in mice (Ma J et al., 2022). In addition, the activation of NRF2 could also attenuate blood brain barrier (BBB) disruption after stroke (Yang et al., 2018). In a recent study, *Nrf2*-knockout was found to exacerbate oxidative stress and apoptosis in brain of hypoxic-ischemic (HI) injury mice, therefore aggravating the brain injury (Zhang Y et al., 2020).

Myricetin, a naturally extracted flavonol compound, is widely found in fruits, vegetables, and berries (Semwal et al., 2016). Studies had shown that it has a variety of biological activities, such as anti-oxidant, anti-inflammatory, and anti-cancer (Salvamani et al., 2014; Stoll et al., 2019). Moreover, due to its excellent safety, myricetin is also used in the research of neonatal disease (Liu et al., 2018; Pluta et al., 2021). It has been reported that myricetin could alleviate intestinal ischemia-reperfusion induced injury through attenuating inflammatory responses and oxidative stress (Sun Y et al., 2018). In addition, myricetin could exert a protective effect on sepsis-associated encephalopathy through modulating inflammasome and apoptotic signaling (Gong et al., 2019). However, there is a lack of research on myricetin in HIE.

So far, there is no report to determine whether myricetin has a neuroprotective role against neonatal HI brain injury. In this study, we would investigate myricetin neuroprotective effects on HI brain injury *in vivo* and *in vitro*, and try to illuminate the potential mechanism. This study may provide a new therapeutic drug for HI brain injury in neonates.

## 2 Materials and methods

### 2.1 Neonatal rat hypoxic-ischemic (HI) brain damage model establishment and drug administration

Sprague-Dawley (SD) rats were purchased from the Animal Center of the Chinese Academy of Sciences (Shanghai, China), and were maintained in the pathogen-free Laboratory Animal Center. Adult rats had access to standard foods and water as well as freely mated to produce offspring for subsequent studies. All experimental operations and animal feeding were approved by the Ethics Committee of Laboratory Animals of Wenzhou Medical University, and were strictly conducted following the Guidelines for the Care and Use of Laboratory Animals. The modified Rice-Vannucci model was used to simulate hypoxic-ischemic (HI) brain damage in male pups 7 days after birth (P7) (Vannucci and Vannucci, 2005). In brief, the P7 pups were anesthetized with isoflurane, and then the left common carotid artery was isolated and ligated within 5 min. After surgery, the pups were returned to the mother to rest fully for 2 h. The rats were then placed in a hypoxic chamber at a constant temperature with 37°C, 92% N_2_, and 8% O_2_ for 2 h. Sham rats were not subjected to arterial ligation and hypoxia. Myricetin was firstly dissolved in DMSO (D8317, Solarbio, Beijing, China), and was then mixed in corn oil. The drug delivery route and dose used in this study were chosen based on previous studies (Sun L et al., 2018; Lin et al., 2020). The pups in HI + Myr group received orally myricetin (HY-15097, MedChemExpress, Monmouth Junction, NJ, United States) at 25 mg/kg dose *via* gavage starting 1 h after HI, and maintained daily administration until the pups were sacrificed. The myricetin used every day should be prepared fresh. *In vivo* experiments, we selected two time-points for parameter detection. At 24 h after HI injury, brain tissues were collected to verify the protective effects of myricetin in the acute stage of injury. Besides, the brain tissues were obtained 7 days after injury to assess the long-term neuroprotective effects of myricetin.

### 2.2 Infarct volume evaluation

The infarct volume of rat brain tissues was quantified by 2,3,5-triphenyltetrazolium chloride (TTC, T8170, Solarbio, Beijing, China) staining (Chumboatong et al., 2020). At 24 h after HI, the rats were deeply anesthetized and perfused to obtain brain tissues, which were frozen at −80°C for several min and then cut into about 2 mm thick coronal sections. The sections were immersed in 1% TTC solution, reacted at 37°C in the dark for 30 min, and fixed in 4% paraformaldehyde solution (P1110, Solarbio, Beijing, China) overnight. The infarct volume of brain tissues was measured using Image J software (National Institutes of Health, Bethessa, MD, United States). The infarct volume (%) = [(contralateral hemispheric volume − ipsilateral hemispheric stained volume)/contralateral hemispheric volume] ×100%.

### 2.3 Measurement of brain edema

As previously described (Jia et al., 2020), we used the dry-wet ratio method to assess the water content of brain tissue. Similarly, we obtained brain tissues at 24 h after HI, and then isolated the left hemisphere and recorded the weight as wet weight. Subsequently, the brain tissues were dried in an oven at 70°C for 72 h for measurement the dry weight. The percentage of moisture content was calculated as [(wet weight − dry weight)/wet weight] ×100%.

### 2.4 Histological staining

At 7 days after HI, the rats were deeply anesthetized and the chest cavity was quickly opened to expose the heart. Then, 20 mL normal saline and 20 mL 4% PFA solution were used for cardiac perfusion at a rate of 10–15 mL/min until their livers were cleared of blood. The rat brains were freshly extracted after decapitation, and then the brain tissues were immediately fixed in 4% PFA for 24 h. After gradient dehydration using ethanol and xylene, the brain tissues were embedded in paraffin and cut into 5 μm thick sections, which were used for subsequent hematoxylin-eosin (HE) (G1120, Solarbio, Beijing, China) and Nissl (G1432, Solarbio, Beijing, China) staining according to the manufacturer’s instructions (Zhu J. J et al., 2021). The images of bright field were obtained under the optical microscope (Nikon Corporatin, Tokyo, Japan), and the number of neurons per mm^3^ was analyzed using Image J software (National Institutes of Health, Bethesda, MD, United States). In addition, 4 images per sample were quantitated (*n* = 4 rats per group).

### 2.5 Immunohistochemistry

As mentioned above, the brain tissues were obtained at 7 days after HI injury and made into paraffin sections. Immunohistochemistry staining was performed as described previously (Wei et al., 2020). The paraffin-embedded sections were dewaxed and hydrated, and then were boiled in preheated sodium citrate buffer (C1010, Solarbio, Beijing, China) for 20 min and cooled to room temperature for antigen retrieval. The endogenous peroxidase was eliminated by 3% H_2_O_2_. After washed with PBS, sections were blocked with 10% goat serum (C0265, Beyotime, Shanghai, China) for 1 h at the room temperature. Then, sections were incubated with primary antibodies (listed in Supplementary Table S1) overnight at 4°C, and followed by the incubation of goat anti-rabbit IgG HRP secondary antibody (1:200, Proteintech, SA00001-2) for 1 h at room temperature. Finally, these slides were visualized with 3, 3′-diaminobenzidine DAB solution under the optical microscope (Nikon Corporatin, Tokyo, Japan).

### 2.6 Immunofluorescence staining

For *in vivo* analysis, we obtained brain tissues 24 h after injury and made them into paraffin-embedded sections. The sections were dewaxed and hydrated for antigen retrieval. *In vitro,* PC12 cells were fixed with 4% PFA solution for 20 min. Then, they were incubated in 10% goat serum with 0.3% Triton X-100 for 1 h at room temperature. After washed with PBS, they were incubated with primary antibodies (listed in Supplementary Table S1) overnight at 4°C, and followed by the incubation of appropriate secondary antibody FITC-conjugated Goat Anti-Mosue IgG (1:200, Proteintech, SA00003-1), Cy3-conjugated Goat Anti-Mouse (1:200, Proteintech, SA00009-1) or Cy3-conjugated Goat Anti-Rabbit (1:200, Proteintech, SA00009-2) for 2 h at room temperature (Zhang W et al., 2020). Finally, the nuclei were stained with DAPI (S2110, Solarbio, Beijing, China), and images were obtained by a fluorescence microscope (Nikon Corporatin, Tokyo, Japan).

### 2.7 TUNEL staining

To analyze apoptosis in brain tissues at 24 h after HI, TUNEL staining was performed using TUNEL Apoptosis Detection Kit (11684817910, Roche, Basel, Switzerland). Briefly, the sections of brain tissues were dewaxed and hydrated, and incubated with the reaction solution in a humid chamber at 37°C for 1 h (Zhu Y et al., 2021). The stained images were captured using a fluorescence microscope, and the number of TUNEL positive cells and DAPI positive staining nuclei were counted using Image J software.

### 2.8 Cell culture and treatment

The differentiated PC12 cells were purchased from the Cell Bank of Chinese Academy of Sciences (Shanghai, China). PC12 cells were maintained in dulbecco’s modified eagle medium (DMEM, Gibco, United States) containing 10% FBS (Gibco, United States) at 37°C in a humidified incubator with 5% CO_2_.

Cellular hypoxia was induced by CoCl_2_ (C8661, Sigma-Aldrich, St. Louis, MO, United States), which is a chemical compound widely used to induce hypoxic-ischemic condition by increasing the generation of ROS (Wang et al., 2000). *In vitro,* PC12 cells were treated with CoCl_2_ for 24 h to induce hypoxia. Myricetin was dissolved in DMSO with a 20 mM stock concentration. According to the preliminary concentration gradient experiments, 200 μM myricetin was selected as the optimal concentration to treat the cells. The myricetin group was pretreated with myricetin 2 h before CoCl_2_ stimulation, while the ML385-treated group was pretreated with NRF2 inhibitor ML385 (HY-100523, MedChemExpress, Monmouth Junction, NJ, United States) (20 μM) for the same time. After the pretreatment, CoCl_2_ was added to each group with myricetin or ML385 according to the experimental requirements.

### 2.9 Cell viability assay

Cell Counting Kit-8 (CCK-8, 40203ES88, Yeasen Biotechnology, Shanghai, China) was used to detect the viability of PC12 cells in different groups. PC12 cells at a density of 1 × 10^4^ cells/well were seeded in 96-well plates. When testing the effect of myricetin, the different doses of myricetin was added into wells 2 h before CoCl_2_ treatment. Then, CoCl_2_ was added to the wells with myricetin. After drug treatment or hypoxia, 10% volume of CCK-8 solution per well was added into each well, which was then maintained in the dark for 30 min at 37°C. The optical density (OD) at 450 nm was measured using a microplate reader (Thermo Fisher Scientific, Waltham, MA, United States).

### 2.10 Annexin V and PI assay

An Annexin V FITC/PI Apoptosis Detection Kit (556547, Becton Dickinson, Franklin Lakes, United States) was used for the detection of cell apoptosis according to the manufacturer’s instructions. Briefly, cells in different groups were harvested by digestion with trypsin (25200072, Gibco, Grand Island, NY, United States), then washed twice with cold PBS, and resuspended in Binding Buffer. The cells were further stained with the mixture of 5 µL FITC Annexin V and 5 µL PI for 15 min at room temperature in the dark (Park et al., 2021). The apoptosis ratio was quantified with a Flow Cytometer (Beckman Coulter, Breya, California, United States).

### 2.11 Reactive oxygen species (ROS) detection

The intracellular ROS levels were determined by a ROS assay kit (S0033S, Beyotime, Shanghai, China) according to the manufacturer’s instructions. In brief, the cells were incubated with diluted DCFH-DA reagent at 37°C for 30 min. Subsequently, the cells were digested with trypsin and harvested (Liao X. et al., 2019). The labeled cells were then detected by the Flow Cytometer (Beckman Coulter, Breya, California, United States).

### 2.12 Mitochondrial ROS detection

A MitoSOX Red Mitochondrial Superoxide Indicator (40778ES50, Yeasen Biotechnology, Shanghai, China) was used to analyze mitochondrial ROS levels. The reagent was diluted with Hank’s equilibating solution (H1025, Solarbio, Beijing, China). Then, cells were incubated with the reagent for 30 min at 37°C in the dark (Zhang et al., 2019). Finally, the nuclei were stained with DAPI, and images were taken under a fluorescence microscope (Nikon Corporatin, Tokyo, Japan).

### 2.13 Western blot

The brain samples at 24 h or 7 days after HI and cells were homogenized with RIPA buffer (R0010, Solarbio, Beijing, China) containing phenylmethane-sulfonyl fluoride (PMSF, P0100, Solarbio, China) and phosphatase inhibitors (P1260, Solarbio, Beijing, China). Nuclear proteins were extracted using a nuclear protein extraction kit (R0050, Solarbio, Beijing, China) according to the manufacturer’s instructions. Protein concentrations were quantified by the BCA kit (ZJ102L, Epizyme Biotech, Shanghai, China). The equal quantities of proteins were separated using 10%–12% sodium dodecyl sulfate-polyacrylamide (SDS-PAGE) gels and transferred to PVDF membranes. The membranes were blocked with 5% skim milk for 3 h at room temperature and incubated with the corresponding primary antibodies (listed in Supplementary Table S1) overnight at 4°C. On the next day, the membranes were washed with Tris-buffered saline with Tween 20 (TBST) and incubated with HRP-Goat Anti-Rabbit IgG (1:5000, Proteintech, SA00001-2) or HRP-Goat Anti-Mouse IgG (1:5000, Proteintech, SA00001-1) secondary antibodies at room temperature for 2 h. The protein bands were visualized using enhanced chemiluminescence (ECL) reagents (MA0186, Meilunbio, Dalian, China) under the ChemiDoc XRS+ Imaging System (Bio-Rad, CA, United States) and analyzed by Image J software. The test indicators of brain samples 24 h after injury include: HIF-1α, Cleaved Caspase-3, BAX, BCL-2, NRF2, KEAP1, HO-1, NQO-1. The test indicators of brain samples 7 days after injury include: MAP-2 and MBP.

### 2.14 Statistical analysis

All experiments were performed at least three times independently. The quantitative data were presented as mean ± SD. Statistical software Graphpad Prism 9.0 (GraphPad Software Inc., CA, United States) was used for statistical analysis. Statistical significance was analyzed by one-way analysis of variance (ANOVA) test followed by Tukey’s test when analyzing more than two groups. Student’s unpaired *t*-test was used for comparisons of two groups. When *p*-value < 0.05, the results were considered statistically significant.

## 3 Results

### 3.1 Myricetin attenuated brain infarction and brain edema

**FIGURE 1:**
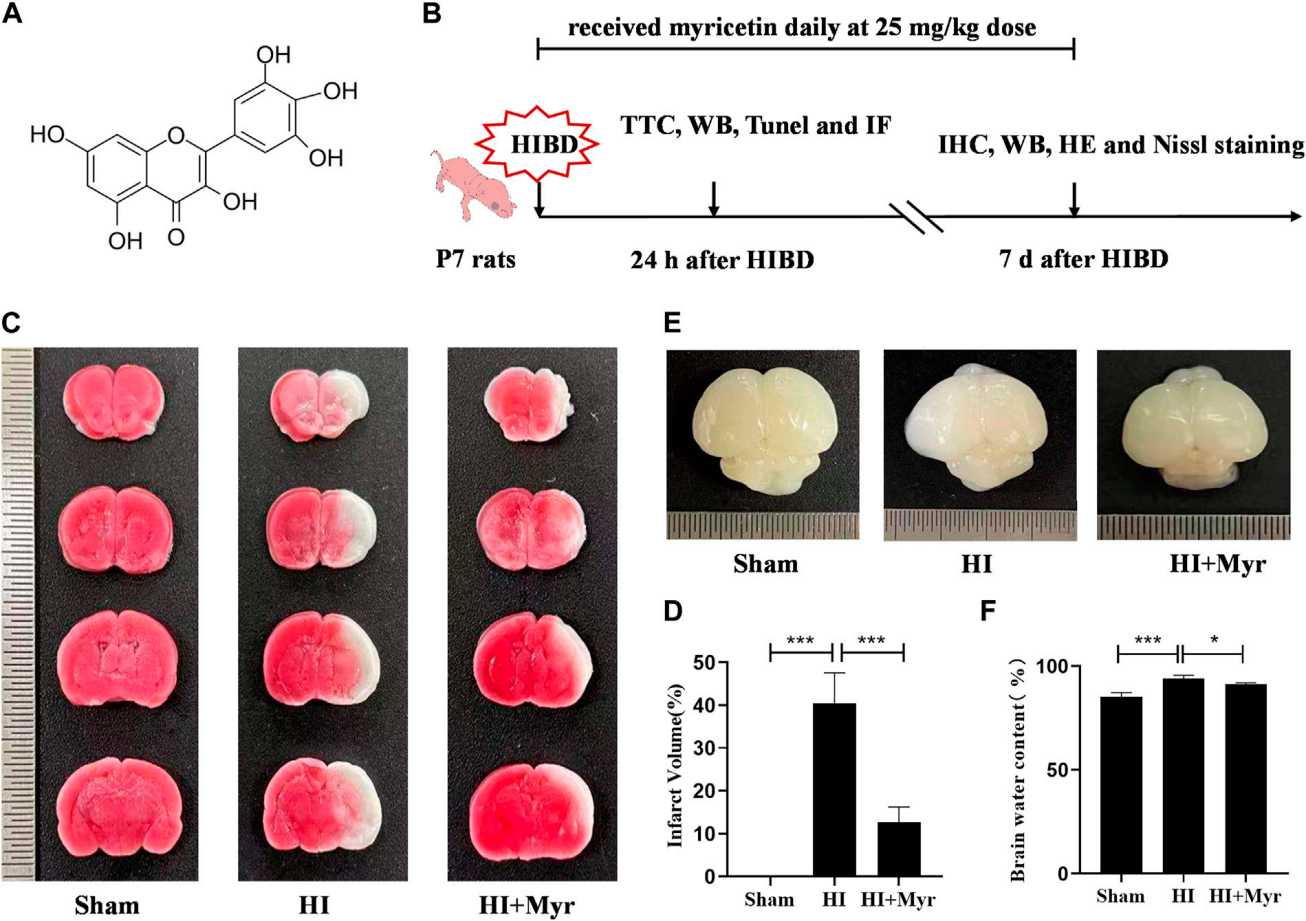
Myricetin attenuated brain infarction and edema. **(A)** The chemical structure of myricetin. **(B)** The timeline of experiment design for *in vivo* experiments. HIBD: hypoxic-ischemic brain damage. **(C)** The representative images of TTC staining of coronary brain sections 24 h after HI brain injury. **(D)** Quantitative analysis of infarct volume based on TTC staining (*n* = 5 per group). **(E)** The representative images of the brain from each group 24 h after HI brain injury. **(F)** The ratio of wet and dry in each group (*n* = 5 per group). The data are presented as mean ± SD. *p < 0.05; ***p < 0.001. Scale bar: 0.5 mm.

The chemical structure of myricetin (Figure 1A) and the timeline of experiment design for *in vivo* experiment (Figure 1B) are shown in this study. To investigate whether myricetin could reduce the damage of HI brain in the acute stage, at the 24 h after HI, TTC staining was performed to assess the brain infarction of rats in Sham, HI, and HI + Myr groups (Figure 1C). The quantitative results of infarct volume showed that there was no infarct in Sham group, and myricetin treatment could significantly reduce the brain infarct volume of HI-induced rats (Figure 1D). Notably, compared with Sham group, there was obvious edema in the ipsilateral cerebral in HI group, while myricetin administration could significantly reduce brain edema of HI-induced rats (Figure 1E). Moreover, the quantitative results of brain water content also showed that the Wet-dry ratio in HI group was significantly higher than that of Sham group, but myricetin intervention could remarkably alleviate this trend for HI-induced rats (Figure 1F). These data suggested that myricetin could effectively alleviate the brain injury in neonatal rats at the acute stage of hypoxic-ischemia.

### 3.2 Myricetin promoted white matter recovery and alleviated brain damage

**FIGURE 2:**
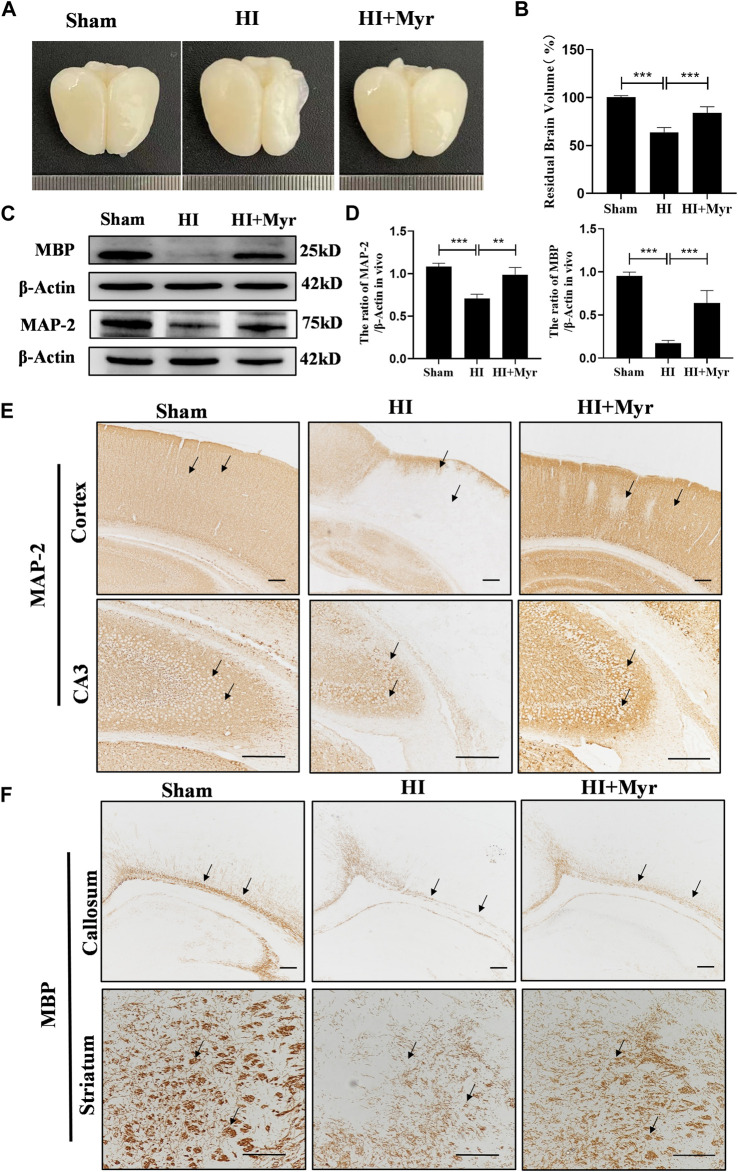
Myricetin alleviated brain atrophy and promoted white matter recovery. **(A)** The representative images of the brain from each group 7 days after HI brain injury. **(B)** Quantitative analysis of residual brain volume (*n* = 6 per group). **(C)** The protein bands of MAP and MBP were detected by Western blot 7 days after HI brain injury. **(D)** Quantitative analysis of Western blot bands (*n* = 4 per group). **(E)** The immunohistochemical staining of MAP-2 in brain cortex and hippocampal CA3 region 7 days after HI brain injury. Black arrows: indicate the density difference of MAP-2. **(F)** The immunohistochemical staining of MBP in brain callosum and striatum region 7 days after HI brain injury. Black arrows: indicate the density difference of MBP. The data are presented as mean ± SD. **p < 0.01, ***p < 0.001. Scale bar: 200 μm.

To further assess the long-term neuroprotective effects of myricetin, we observed the brain anatomy at 7 days after HI (Figure 2A). The quantitative results of residual brain volume showed that compared with Sham group, the brain atrophy was obvious in HI group, and myricetin treatment could significantly alleviate the degree of brain atrophy in HI-induced rats (Figure 2B). Microtubule-associated protein-2 (MAP-2) is the main cytoskeletal regulator within neuronal dendrites that serves as a robust somatodendritic marker (DeGiosio et al., 2022). Myelin basic protein (MBP) is a key component of myelin sheath in the central nervous system that serves as an oligodendrocyte marker (Harauz et al., 2004). Western blot (Figure 2C) and immunohistochemical staining of MAP-2 (Figure 2E) in brain cortex and hippocampal CA3 region and MBP (Figure 2F) in callosum and striatum regions were performed to investigate whether myricetin could accelerate axonal repair and remyelination 7 days post-HI injury. The expression trends of MAP-2 and MBP proteins by Western blot were consistent with that of immunohistochemical staining. In addition, the quantitative results of Western blot showed that the expression of MAP-2 and MBP were significantly lower in HI group than that of Sham group, whereas myricetin could significantly reverse this trend (Figure 2D).

**FIGURE 3:**
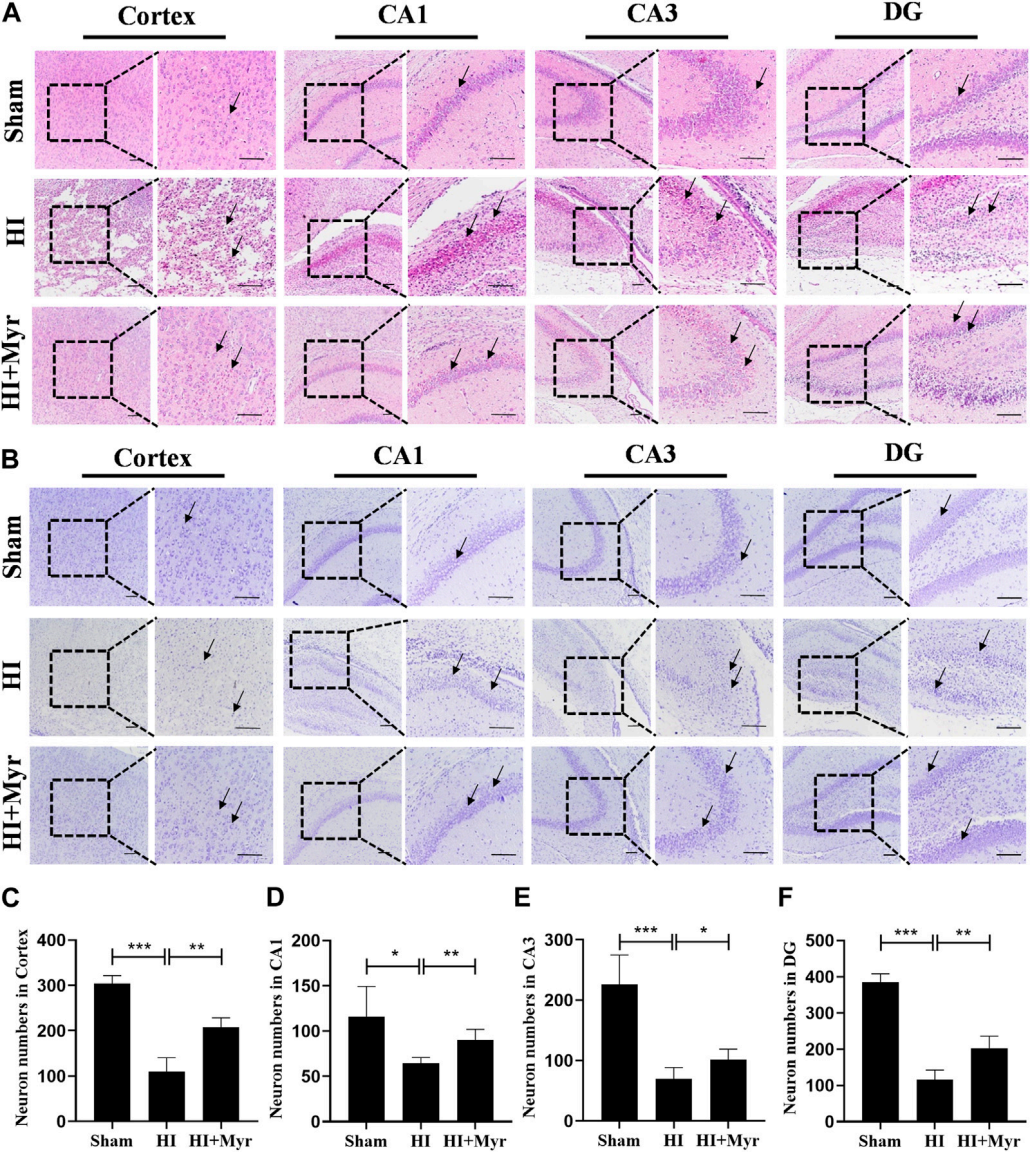
Myricetin ameliorated the tissue structural damage and reduced the loss of neurons. **(A)** The representative images of HE staining in brain cortex and hippocampal CA1, CA3, DG regions 7 days after HI brain injury. Black arrows: indicate the morphology of brain tissue and cells. **(B)** The representative images of Nissl staining in brain cortex and hippocampal CA1, CA3, DG regions 7 days after HI brain injury. Black arrows: indicate the Nissl body integrity. **(C)** Quantification analysis of neuron numbers in brain cortex (*n* = 4 per group). **(D)** Quantification analysis of neuron numbers in hippocampal CA1 region (*n* = 4 per group). **(E)** Quantification analysis of neuron numbers in hippocampal CA3 region (*n* = 4 per group). **(F)** Quantification analysis of neuron numbers in hippocampal DG region (*n* = 4 per group). The data are presented as mean ± SD. *p < 0.05, **p < 0.01, ***p < 0.001. Scale bar: 100 μm.

Moreover, the morphology of brain tissue at 7 days after HI was observed by HE staining (Figure 3A). Compared with sham group, the HE staining of brain cortex and hippocampal CA1, CA3, DG regions in HI group showed that the cells were extensively damaged, which were characterized by neuronal shrinkage and nuclear chromatin condensation. However, myricetin could significantly ameliorate these phenomena. Meanwhile, we measured the neuron numbers in those regions by Nissl staining (Figure 3B). The quantitative results revealed that compared with Sham group, a considerable of neurons were lost in the brain cortex (Figure 3C) and hippocampal CA1 (Figure 3D), CA3 (Figure 3E), DG (Figure 3F) regions of HI-induced rats, and these pathological changes were significantly alleviated by myricetin treatment. These results indicated that myricetin could reduce neuronal loss, promote morphological recovery, stabilize microtubule function, and promote myelination after HI brain injury in the long-term stage.

### 3.3 Myricetin inhibited cell apoptosis induced by HI brain injury

**FIGURE 4:**
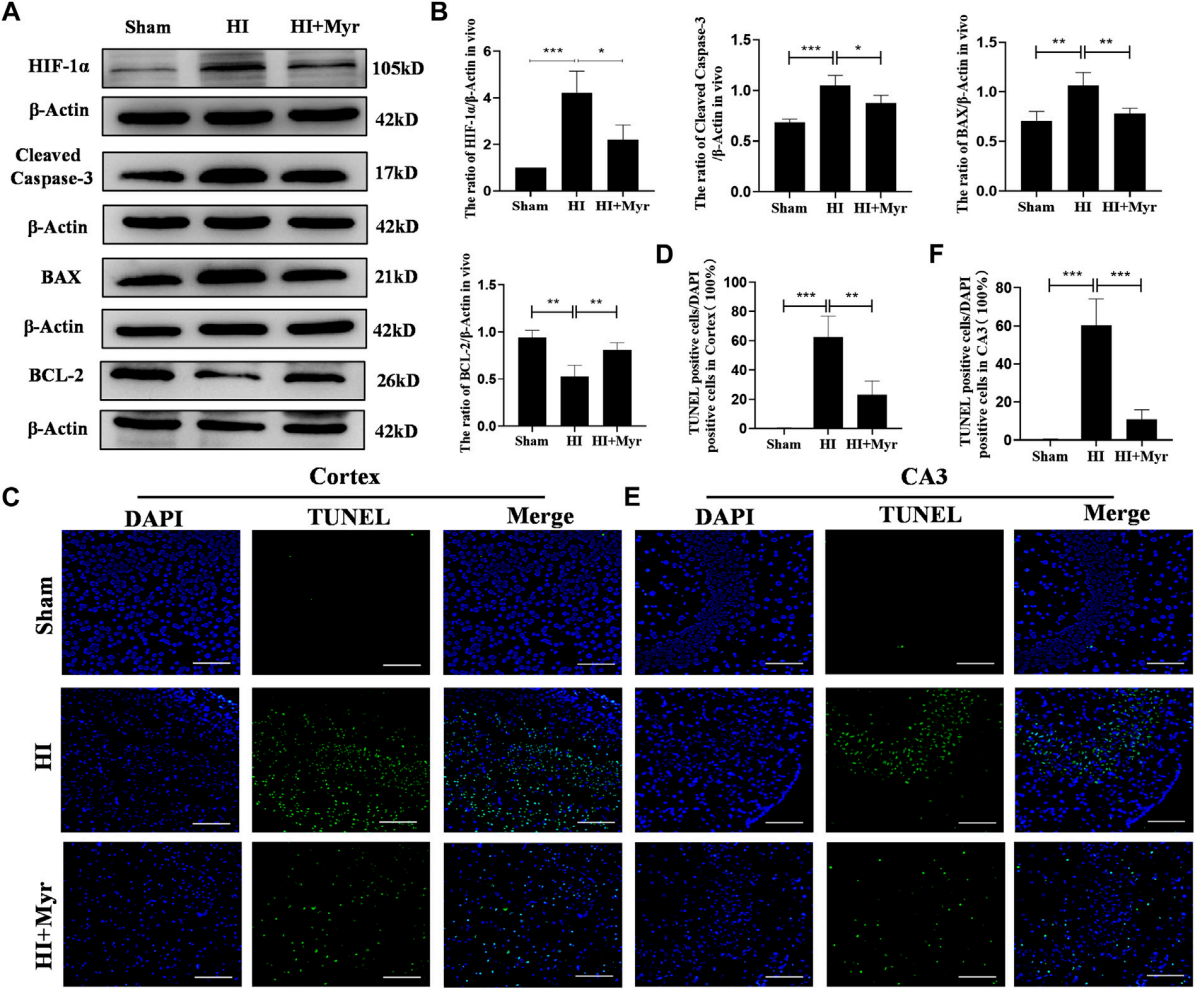
Myricetin reduced apoptosis induced by hypoxic-ischemic brain injury. **(A)** The protein bands of HIF-1α, Cleaved Caspase-3, BAX, and BCL-2 were detected by Western blot 24 h after HI brain injury. **(B)** Quantitative analysis of Western blot bands (*n* = 4 per group). **(C)** The representative images of TUNEL staining in brain cortex 24 h after HI brain injury. Nucleus were stained with DAPI (blue). **(D)** Quantitative analysis of TUNEL staining in brain cortex (*n* = 4 per group). **(E)** The representative images of TUNEL staining in hippocampal CA3 region 24 h after HI brain injury. **(F)** Quantitative analysis of TUNEL staining in hippocampal CA3 region (*n* = 4 per group). The data are presented as mean ± SD. *p < 0.05, **p < 0.01, ***p < 0.001. Scale bar: 100 μm.

In order to investigate whether myricetin exists anti-apoptosis effect on rat brain tissues, at 24 h after HI, Western blot was performed to detect the expression of apoptosis-related proteins in different groups (Figure 4A). HIF-1α, a crucial transcription factor in the cellular response to hypoxia, which is involved in diverse processes, such as angiogenesis, cell proliferation, and apoptosis/survival (Semenza, 2012). The quantitative results of Western blot showed that compared with Sham group, the expressions of HIF-1α, Cleaved Caspase-3, and BAX were significantly upregulated as well as the level of BCL-2 was significantly downregulated in HI group, and myricetin treatment could dramatically reverse these trends in HI induced rats (Figure 4B). Meanwhile, the TUNEL staining of brain cortex (Figure 4C) and hippocampal CA3 region (Figure 4E) areas was conducted to further assess the anti-apoptosis effect of myricetin. The quantitative data of TUNEL staining displayed that there was almost no TUNEL-positive cell in brain cortex and hippocampal CA3 region of Sham group, but the proportions of TUNEL-positive cells were noticeably increased in brain cortex (Figure 4D) and hippocampal CA3 region (Figure 4F) areas of HI group, which could be reversed by myricetin treatment. Those findings suggested that myricetin might exert anti-apoptosis effect to alleviate HI induced brain injury through inhibiting anti-oxidant transcription factor HIF-1α expression.

### 3.4 Myricetin reduced glial activation and changed the protein expression of NRF2 pathway

**FIGURE 5:**
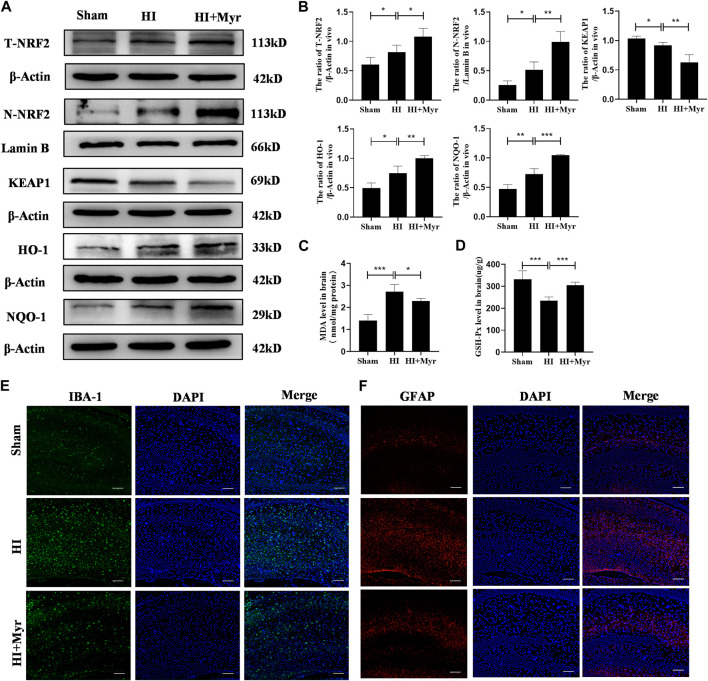
Myricetin attenuated excessive oxidative stress (ROS) and reduced glial activation through NRF2 pathway. **(A)** The protein bands of T-NRF2, N-NRF2, KEAP1, HO-1, and NQO-1 were evaluated by Western blot in brain tissues 24 h after HI injury. **(B)** Quantitative analysis of Western blot bands (*n* = 4 per group). **(C)** The MDA level in brain tissues 24 h after HI brain injury (*n* = 5 per group). **(D)** The GSH-px level in brain tissues 24 h after HI brain injury (*n* = 5 per group). **(E)** The representative immunofluorescence staining images of IBA-1 (green). Nucleus were stained with DAPI (blue). **(F)** The representative immunofluorescence staining images of GFAP (red). The data are presented as mean ± SD. *p < 0.05, **p < 0.01, ***p < 0.001. Scale bar: 100 μm.

The oxidative stress is a well-known mechanism for the pathogenesis of hypoxia-ischemia brain damage (Vasiljevic et al., 2012). In addition, it has been reported that myricetin could produce potent anti-oxidant effects and effectively activate nuclear factor E2-related factor 2 (NRF2) (Liao H. H. et al., 2019). Thus, the expression levels of NRF2 protein in different groups were detected by Western blot (Figure 5A). The quantitative results of Western blot showed that the expressions of total NRF2 (T-NRF2) and nuclear NRF2 (N-NRF2) in HI group were higher than that of Sham group, and myricetin administration further upregulated the levels of T-NRF2 and N-NRF2 in HI-induced rats. Specifically, the ratio of T-NRF2/β-Actin increased from 0.82 ± 0.12 in HI group to 1.08 ± 0.14 in HI + Myr group, and the ratio of N-NRF2/Lamin B increased from 0.51 ± 0.14 to 0.99 ± 0.17. Meanwhile, myricetin treatment also increased HO-1 and NQO-1 expressions in HI-induced rats, which depended on the activity of transcriptional activator NRF2, and downregulated KEAP1 expression (Figure 5B). Moreover, compared with HI group, myricetin also downregulated MDA content (Figure 5C) and increased the content of GSH-Px (Figure 5D) in HI-induced rats.

The previous study had confirmed that ROS is also involved in the activation of glia cells, and the NRF2 signaling pathway plays a critical role in the regulation of oxidation/anti-oxidant imbalance in glia cells (Zhu et al., 2022). Therefore, immunofluorescence staining was conducted to detect the expression of IBA-1 that is a surface maker of activated microglia (Figure 5E) and GFAP that is a maker of reactive astrogliosis (Figure 5F) to explore whether myricetin could reduce the activation of glia cells. It was shown that compared with Sham group, the microglia and astrogliosis were markedly activated in HI group, and this trend could be reversed by myricetin administration. These data suggested that myricetin might play neuroprotective effects for HI injury through NRF2 pathway.

### 3.5 Myricetin alleviated CoCl_2_-induced PC12 injury

**FIGURE 6:**
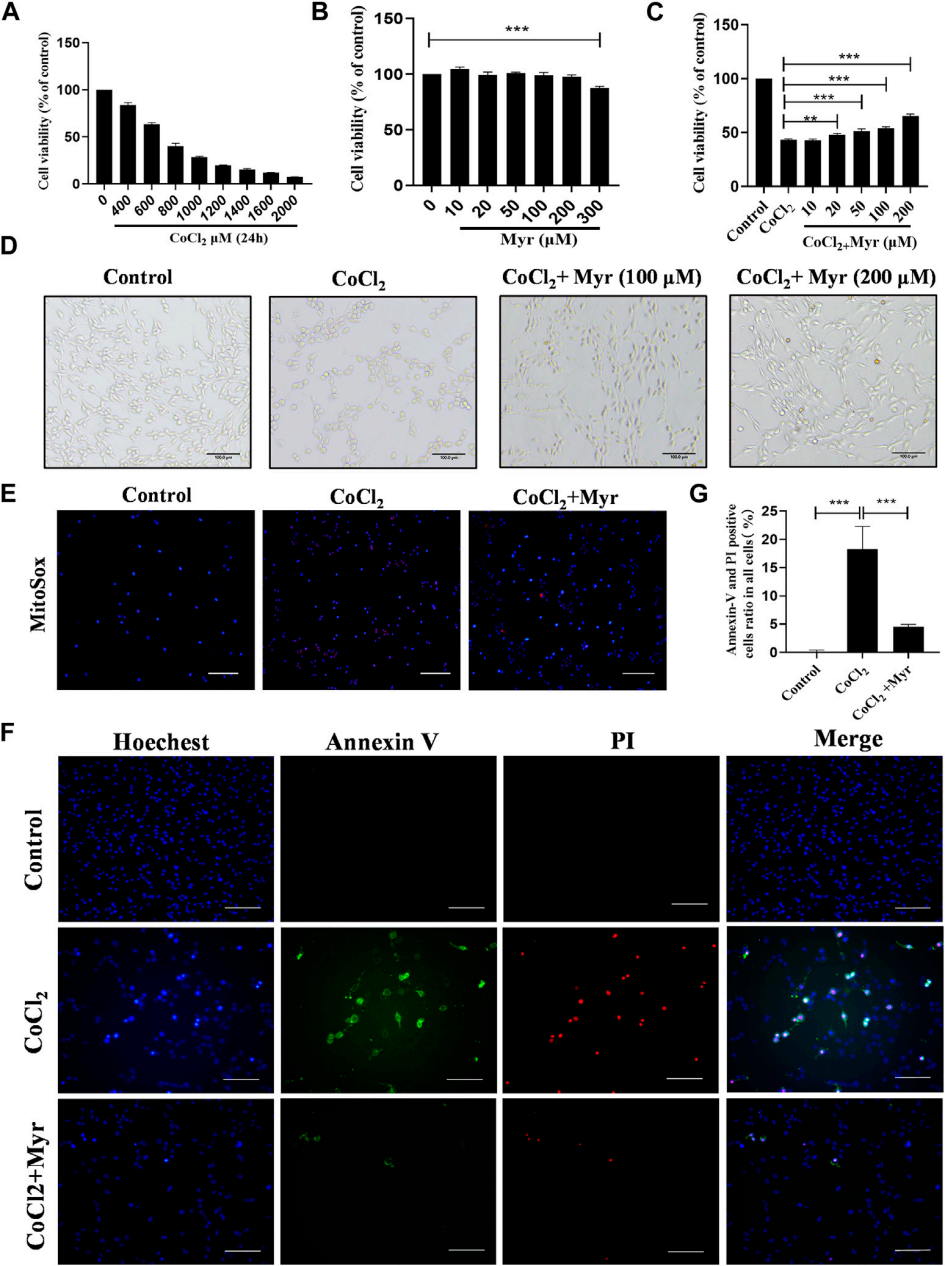
Myricetin alleviated CoCl_2_-induced injury and mitochondrial ROS production in PC12 cells. **(A)** The cell viability of PC12 treated with different dose CoCl_2_ for 24 h using CCK-8 (*n* = 4 per group). **(B)** The safe dose ranges of myricetin to maintain cell viability was determined by CCK-8 assay (*n* = 4 per group). **(C)** The dose-dependent effect of myricetin on PC12 cell viability after CoCl_2_ injury (*n* = 4 per group). **(D)** The effects of myricetin at different doses on morphology of PC12 cells after CoCl_2_ injury. **(E)** The generation of Mitochondrial ROS in PC12 was detected by MitoSOX (red) and DAPI (blue) staining. **(F)** The images of Annexin V FITC (green) and PI (red) co-staining to detect apoptosis. The nuclei were labeled with Hoechest (blue). **(G)** Quantitative analysis of Annexin V and PI positive cells (*n* = 4 per group). The data are presented as mean ± SD. **p < 0.01, ***p < 0.001. Scale bar: 200 μm in **(E)**; 100 μm in **(F)**.

To further ascertain the potential *in vivo* mechanism mentioned above, CoCl_2_ was used to simulate HI model for PC12 cells *in vitro*. The toxic effect of CoCl_2_ on PC12 cell viability was assessed by CCK-8 assay. The results showed that PC12 cells treated with various concentrations of CoCl_2_ (400–2000 μM) for 24 h displayed the dose-dependent decreasing cell viability compared with 0 μM. In addition, the viability of PC12 cells exposed to 800 μM CoCl_2_ for 24 h was decreased to 40.4% ± 2.93% (Figure 6A). So, the concentrations of 800 μM CoCl_2_ was chosen for the subsequent experiments. Then, the cytotoxicity of myricetin on PC12 cells was also detected by CCK-8 assay. Myricetin (10–200 μM) had no obvious adverse reaction on PC12 cell viability, while the cell viability decreased at dose of 300 μM (Figure 6B). PC12 cells were pretreated with various concentrations myricetin (10–200 μM) for 2 h before 800 μM of CoCl_2_ treatment, and the cell viability increased and reached the optimal at dose of 200 μM (Figure 6C). The images of PC12 cells were captured immediately after CoCl_2_ for 24 h in each group. Shrinkage and turn round of PC12 cells in CoCl_2_ group were observed through the optical microscope. Myricetin treatment could alleviate these phenomena and the most obvious effect was observed at dose of 200 μM (Figure 6D).

In addition, the MitoSOX staining was performed to analyze the ROS level in each group. The results showed that CoCl_2_ could significantly induce the production of mitochondrial ROS (red staining), but myricetin intervention could markedly reduce the mitochondrial ROS level in CoCl_2_-induced PC12 cells (Figure 6E). Meanwhile, the co-staining of Annexin V and PI was used to detect CoCl_2_-induced PC12 apoptosis *in vitro* (Figure 6F). Cells in early apoptosis were PI negative and Annexin V positive, while in late apoptosis were both Annexin V and PI positive. The quantitative results showed that there was almost no Annexin V or PI positive cell in Control group, but CoCl_2_ could notably increase the production of Annexin V or PI positive cells, and myricetin treatment could significantly reverse this trend (Figure 6G). These data suggested that myricetin could alleviate CoCl_2_-induced PC12 cell injury through inhibiting mitochondrial ROS production and apoptosis.

### 3.6 Myricetin alleviated CoCl_2_-induced PC12 injury by activating NRF2 pathway

**FIGURE 7:**
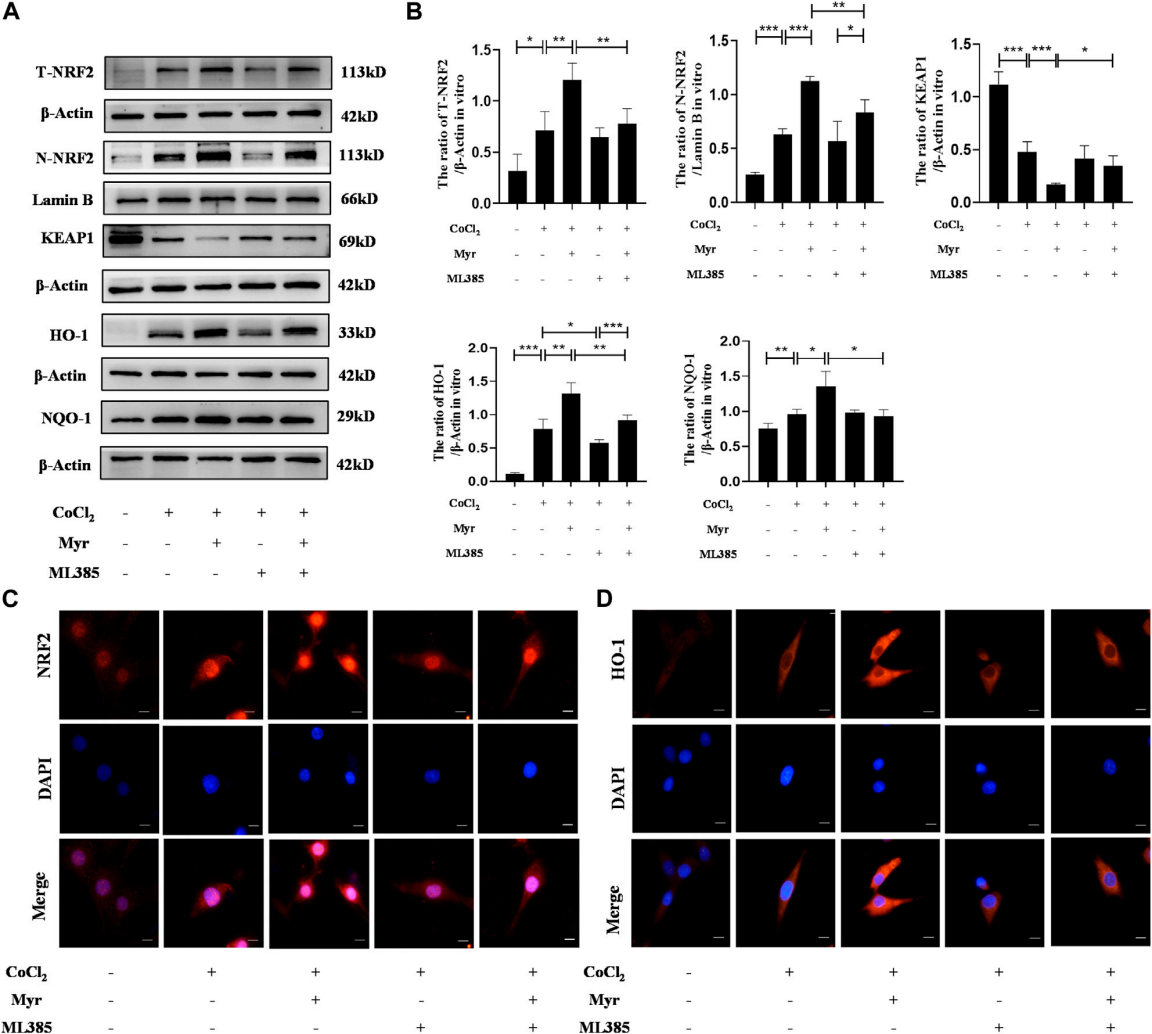
Myricetin exerted neuroprotection through the activation of NRF2 pathway. **(A)** The protein bands of T-NRF2, N-NRF2, KEAP1, NQO-1, and HO-1 were detected by Western blot in PC12 from each group. **(B)** Quantitative analysis of Western blot bands (*n* = 4 per group). **(C)** The representative immunofluorescence staining images of NRF2 (red) in PC12 from each group. Nucleus were stained with DAPI (blue). **(D)** The representative immunofluorescence staining images of HO-1 (red) in PC12 from each group. The data are presented as mean ± SD. *p < 0.05, **p < 0.01, ***p < 0.001. Scale bar: 10 μm.

To further explore the potential mechanism of myricetin on CoCl_2_-induced PC12 injury, the NRF2 typical antagonist ML385 was used to assess the relative proteins expressions of NRF2 pathway by Western blot (Figure 7A). The quantitative results showed that the levels of total NRF2 (T-NRF2) and nuclear NRF2 (N-NRF2) were higher in CoCl_2_-induced PC12 with the ratios of 0.71 ± 0.19 and 0.63 ± 0.05. In addition, myricetin treatment further increased the levels of T-NRF2 and N-NRF2 in CoCl_2_-induced PC12 and the ratio increased to 1.21 ± 0.16 and 1.12 ± 0.04. Meanwhile, myricetin treatment also increased HO-1 and NQO-1 levels but decreased KEAP1 level in CoCl_2_-induced PC12. Moreover, ML385 could significantly reverse the effect of myricetin in CoCl_2_-induced PC12. The quantitative results showed that after combined use of myricetin and ML385, the ratio of T-NRF2/β-Actin decreased to 0.78 ± 0.15 and the ratio of N-NRF2/Lamin B decreased to 0.83 ± 0.12 (Figure 7B). In addition, the immunofluorescence staining results of NRF2 (Figure 7C) and HO-1 (Figure 7D) were consistent with the Western blot. These data suggested that myricetin could alleviate CoCl_2_-induced PC12 injury through activating NRF2 pathway.

### 3.7 Myricetin reduced oxidative stress and apoptosis in CoCl_2_-induced PC12 by activating NRF2 pathway

**FIGURE 8:**
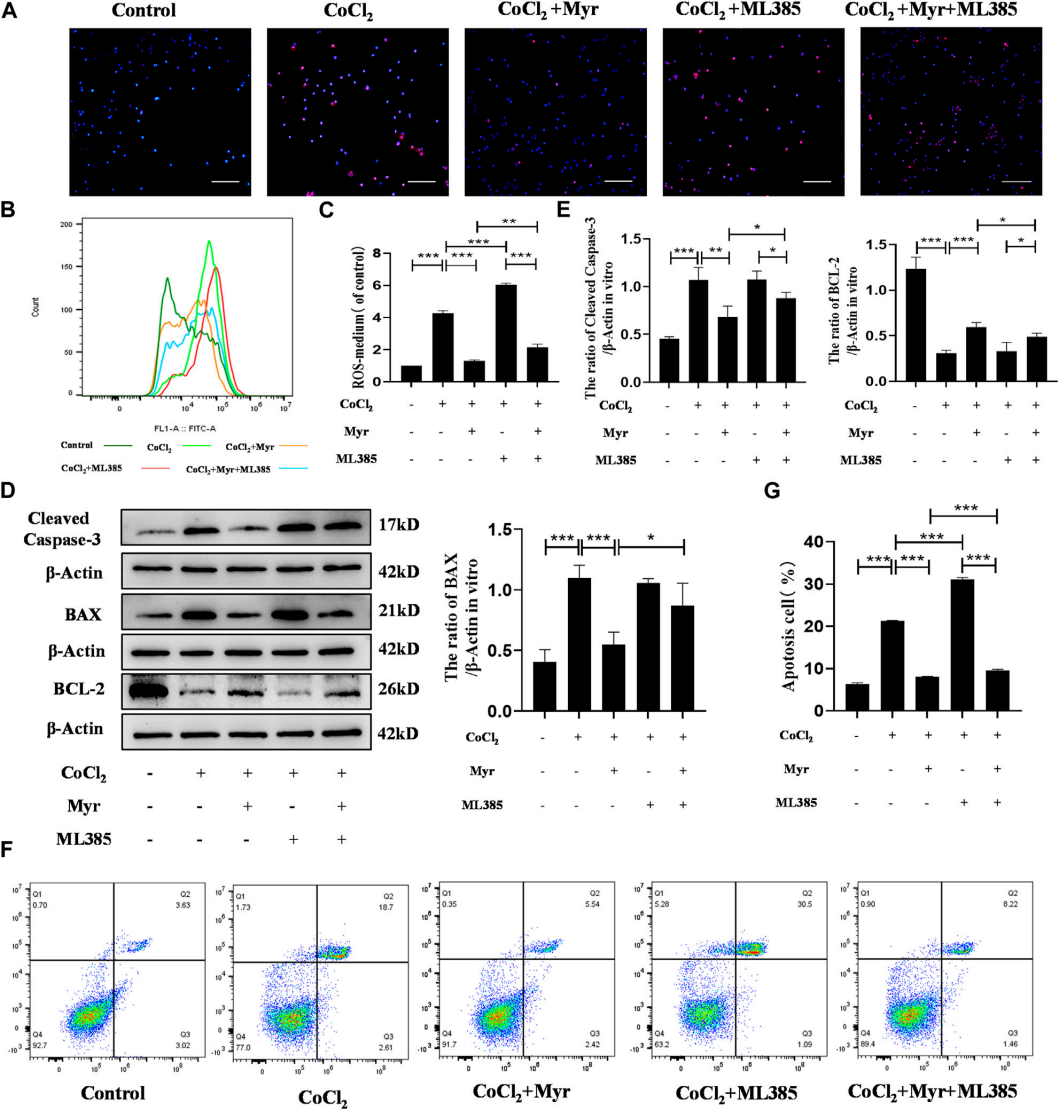
Myricetin reduced ROS production and apoptosis in PC12 cells through activating NRF2 pathway. **(A)** The generation of mitochondrial ROS in each group was detected by MitoSOX (red) and DAPI (blue) staining. **(B)** The cell ROS in each group was analyzed using flow cytometry after DCF-DA staining. **(C)** Quantitative analysis of flow cytometry after DCF-DA staining (*n* = 3 per group). **(D)** The protein bands of Cleaved Caspase-3, BAX, and BCL-2 were detected by Western blot. **(E)** Quantitative analysis of Western blot bands (*n* = 4 per group). **(F)** The apoptosis in each group was analyzed using flow cytometry after Annexin V FITC and PI co-staining. **(G)** Quantitative analysis of flow cytometry after Annexin V FITC and PI co-staining (*n* = 4 per group). The data are presented as mean ± SD. *p < 0.05, **p < 0.01, ***p < 0.001. Scale bar: 200 μm.

In order to further demonstrate that myricetin could alleviate CoCl_2_-induced PC12 injury *in vitro* through activating NRF2 pathway, the NRF2 inhibitor ML385 was used to detect the oxidative stress and apoptosis in different groups. The MitoSOX staining showed that myricetin administration could significantly decrease the mitochondrial ROS level in CoCl_2_-induced PC12 cells, but the effect of myricetin combined with ML385 was relatively week (Figure 8A). In addition, the flow cytometry was used to detect the cell ROS levels in different groups (Figure 8B). The quantitative results showed that CoCl_2_ could obviously increase the level of cell ROS, but myricetin intervention could markedly reduce the cell ROS level in CoCl_2_-induced PC12 cells. Similarly, ML385 could reverse myricetin effect on CoCl_2_-induced PC12 (Figure 8C). Moreover, Western blot was conducted to detect the apoptosis-related protein expressions in different groups (Figure 8D). The quantitative results showed that CoCl_2_ could significantly upregulate the expressions of Cleaved Caspase-3 and BAX and downregulate the expression of BCL-2, but myricetin treatment could dramatically reverse these trends in CoCl_2_-induced PC12 cells. Similarly, combined with ML385, the effect of myricetin was relatively week (Figure 8E). The flow cytometry of Annexin V and PI assay was performed to detect the CoCl_2_-induced apoptosis of PC12 in different groups (Figure 8F). The percentages of apoptosis cells were calculated from the early apoptosis (Q3) and late apoptosis (Q2). The quantitative result was consistent with the above cell ROS results (Figure 8G). These findings further indicated that myricetin could attenuate CoCl_2_-induced cell oxidative stress and apoptosis to exert protective roles through NRF2 pathway.

**FIGURE 9:**
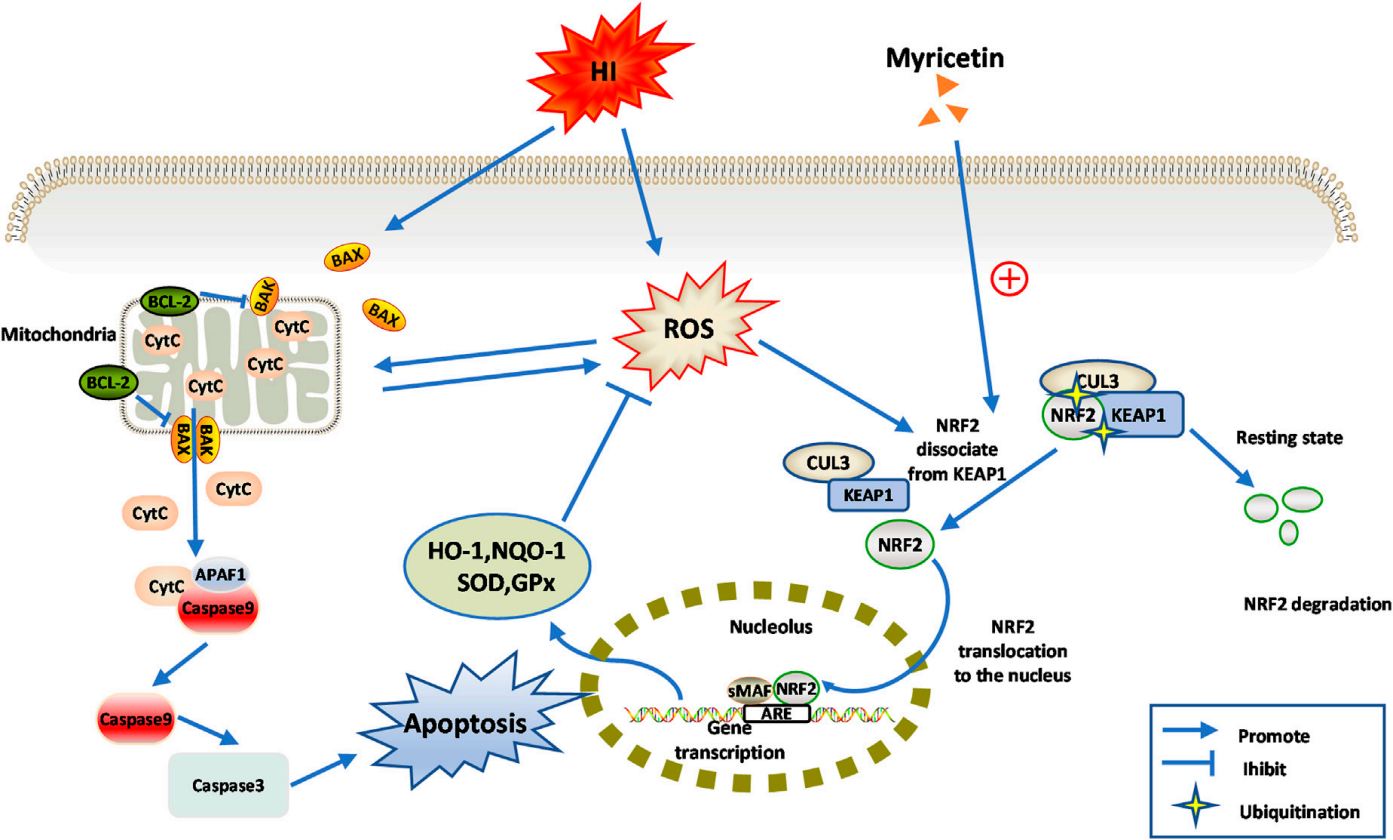
Diagram of the potential mechanisms involved the neuroprotective effects of myricetin against HI-induced brain injury or CoCl_2_ induced PC12 injury.

Based on the above findings, we carefully speculated that myricetin might attenuate oxidative stress and apoptosis through NRF2 pathway, thereby alleviating neonatal HI injury, which suggested that myricetin might be a promising therapeutic agent for HIE. The detail diagram of mechanisms involved in the effects of myricetin on HI injury was shown in Figure 9.

## 4 Discussion

Although the medical advances have improved the treatment of neonatal brain injury, hypoxic-ischemic encephalopathy (HIE) remains a concern. Hypothermia is the current standard treatment method for HIE, but it has limited effects on patients with moderate and severe injury, and has a narrow treatment time window (Nair and Kumar, 2018). Thus, there is an urgent need to search more effective therapy approaches for HIE. Currently, study had identified oxidative stress and apoptosis as the important factors for HIE pathological injury (Huang et al., 2019). In this study, we tried to explore the neuroprotective effects of myricetin on HI brain injury *in vivo* and *in vitro*, and illuminate the potential mechanism.

Myricetin, a polyhydroxyflavonol compound isolated from myria rubra tree bark, has attracted extensive attention due to its beneficial pharmacological effects (Song et al., 2021). In recent years, several studies had shown that myricetin had anti-oxidant, anti-bacterial, anti-inflammatory, immunomodulatory, and anti-cancer effects (Alcaraz and Ferrandiz, 2020; Taheri et al., 2020). Myricetin can alleviate neuroinflammation caused by LPS (Jang et al., 2020), reduce diabetic neuropathy (Ma S et al., 2022), and improve learning and memory functions in Alzheimer’s disease (Ramezani et al., 2016), indicating that it has neuroprotective effects. However, there is no report to demonstrate whether myricetin has the protective effects on HI-induced brain damage. Herein, we tried to verify the protective roles of myricetin on HI-induced brain injury. Our results *in vivo* showed that myricetin could not only alleviate acute brain injury caused by HI, but also protect the structural integrity of brain tissues in long-term. Our data *in vitro* displayed that myricetin could promote cell viability and reduce cell apoptosis as well as ROS production in CoCl_2_-induced PC12. In addition, both *in vivo* and *in vitro* results showed that myricetin could upregulate the expressions of NRF2 pathway related indicators, which could be reversed by ML385, the specific inhibitor of NRF2. Thus, we speculated that NRF2 signaling pathway might be involved in the protective mechanisms of myricetin in HI brain injury.

The pathogenic processes of HI injury are complex, and its pathological mechanism is classified into three phases. Initially, the reduced cerebral blood flow results in anaerobic metabolism, accumulation of extra-cellular amino acids, and cell swelling (Greco et al., 2020). Then, reperfusion leads to mitochondrial dysfunction, large amounts of ROS production, and the releases of intracellular calcium as well as various inflammatory factors (Arteaga et al., 2017). The imbalance between ROS and antioxidants leads to oxidative stress (Salim, 2017), which causes protein oxidation, lipid peroxidation, and DNA destruction (Hassan et al., 2022). The neonatal brains are more susceptible to oxidative stress due to high rate of oxygen consumption, rich in unsaturated fatty acids and metal ions, and weak antioxidant capacity (Qin et al., 2019). *In vivo* HI-induced brain injury model, the content of MDA was increased, but the level of antioxidant GSH-Px. Mitochondria plays a key role in the process of oxidative stress, and is the main site for ROS production (Yildiz et al., 2017). The results of MitoSOX fluorescence staining and ROS flow cytometry showed that the mitochondria ROS and the total intracellular ROS were significantly increased after HI injury. Meanwhile, myricetin could upregulate the antioxidant level and reduce the levels of both the mitochondrial and total intracellular ROS. Moreover, cells also can induce endogenous antioxidant production to limit free radical reactions (Patel, 2016).

NRF2 (nuclear factor-E2-related factor 2) is a transcription factor that can induce a set of anti-oxidants and detoxication enzymes expressions, and can be regulated by KEAP1 (Sies et al., 2017). In basal condition, NRF2 is present in cytoplasm bond to the KEAP1 which acts as a substrate adaptor for CUL3 ubiquitin ligase-mediated proteasomal degradation of NRF2 (Todorovic et al., 2016). Thus, NRF2 protein degrades rapidly in normal cells, and remains low in abundance. In response to oxidative stress, the structure of KEAP1/NRF2/CUL3 complex begins to change, thereby inhibiting NRF2 ubiquitination to cause NRF2 translocation into the nucleus (Ma, 2013). After entering the nucleus, NRF2 can be heterodimerized with small MAF proteins (sMAF), and then bind to anti-oxidant response element (ARE) to promote the transcription of target genes. Both HO-1 and NQO-1 are belonged to the downstream target genes of NRF2 (Tonelli et al., 2018), which play the anti-oxidative stress role. Consistent with the results of previous studies (Xie et al., 2021; Li et al., 2022), our *in vivo* and *in vitro* experiment results showed that the nuclear translocation of NRF2 was promoted in the acute phase of HI induced injury, and the protein expressions of NRF2, HO-1, and NQO-1 were upregulated. Zhang et al. (2016) found that myricetin could alleviate cuprizone induced behavioral dysfunction and demyelination in mice by activating NRF2. Similarly, our study found that NRF2 was significantly activated to undergo nuclear transfer after myricetin intervention, and the expressions of endogenous anti-oxidant enzymes were further increased. In addition, NRF2 can also regulate the levels of anti-oxidant enzymes, such as SOD, CAT, and GSH-Px. Myricetin could increase the amount of these enzymes, which is also consistent with experimental results mentioned above. The role of glial cells in HIE should not be ignored, and NRF2 is one of the important factors in regulating the activity of glial cells. Liu et al. (2020) had identified that compared with WT mice, HI could trigger more obvious activation of microglia, and evoke more severe proliferation of astroglia in *Nrf2* ^−/−^ mice. In our study, myricetin could alleviate glial activation, so we hypothesized that it might also act through activating NRF2 pathway. To further verify our hypothesis, the NRF2 specific inhibitor ML385 was used in this study. As expected, ML385 intervention significantly counteracted the anti-oxidant and anti-apoptosis effects of myricetin, reduced NRF2 nuclear translocation, and downregulated anti-oxidant enzyme activities.

Mitochondria are the main sites for ROS production, but they are also the main targets for ROS attack, forming a vicious cycle between each other (Jezek et al., 2018). The accumulation of ROS caused by hypoxia can lead to the decrease of mitochondrial membrane potential and the increase of membrane permeability. Then, cytochrome C is released into the cytoplasm and initiates the apoptotic Caspase-9/3 activation cascade to fragment DNA (Zhao et al., 2016). This process can be regulated by BCL-2 family proteins, including anti-apoptotic proteins, such as BCL-2, BCL-xl, and MCL-1 as well as pro-apoptotic proteins, such as BAX, BAD, etc. (Yang et al., 2020; Chen et al., 2022). During the pathological development of neonatal HI brain injury, apoptosis is the more prevalent mode of death, and is more severe in the immature brain (Lai and Yang, 2011). Therefore, apoptosis intervention will effectively improve the prognosis of HIE. Zhang et al. (2018) verified that myricetin could attenuate cardiomyocyte apoptosis induced by LPS. In the present study, myricetin treatment could significantly downregulate the expressions of BAX and Cleaved Caspase-3, increase the level of BCL-2, and reduce the numbers of TUNEL-positive cells in neonatal HI brain of rats. The same effects were observed *in vitro* experiments. There are accumulating evidences that NRF2 is involved in the regulation of apoptosis, and NRF2-deficient cells show increased spontaneous apoptosis (Ma, 2013; Lv et al., 2020). Thus, we wondered whether myricetin could also act against apoptosis through activation of NRF2 pathway. Our results showed that compared with myricetin treatment, ML385 intervention could increase the number of apoptosis and upregulate the expressions of apoptosis proteins in CoCl_2_-induced PC12 cells. On the one hand, we hypothesized that myricetin can reduce apoptosis by alleviating oxidative stress, but further studies are needed to explore the underlying mechanism.

In this study, the current results suggested that myricetin played a neuroprotective role in the neonatal rat brain after HI injury by alleviating oxidative stress and apoptosis through activating NRF2 signaling pathway. However, there are limitations of our study. Firstly, ML385 was only used to detect the protective effect of myricetin *in vitro*, and the further studies on inhibiting NRF2 *in vivo* are needed. In addition, we need to measure the motor learning ability of rats in each group to reflect the long-term protective effect of myricetin. Moreover, we should also analyze the kinetics of NRF2 decay to determine whether myricetin affects NRF2 protein stability, which will help to further elucidate the mechanism of myricetin. Finally, although PC12 cells are widely used in nervous system studies, in order to simulate HI conditions *in vivo*, it is better to extract primary cortical nerve cells for oxygen-glucose deprivation experiment.

## 5 Conclusion

In summary, the oral administration of myricetin played neuroprotective roles in HI brain damage rats which could significantly reduce the infarct volume and improve long-term neurological prognosis. Furthermore, myricetin intervention could inhibit oxidative stress and apoptosis. Then, the anti-oxidative stress and anti-apoptosis effects of myricetin might be involved in NRF2 signaling pathway. Taken together, our results suggested that myricetin might serve as a potential therapeutic drug for HI brain injury.

## Data Availability

The original contributions presented in the study are included in the article/Supplementary Material, further inquiries can be directed to the corresponding authors.
