# Supplementary material for: Myricetin attenuates hypoxic-ischemic brain damage in neonatal rats via NRF2 signaling pathway
Source: Front Pharmacol. 2023 Mar 8;14:1134464. doi: 10.3389/fphar.2023.1134464 (PMC10031108; doi:10.3389/fphar.2023.1134464)
Supplement: Supplementary file 1 [file Table1.pdf]

Supplemental Table 1. the antibodies used in this study.

Antibody	Species	Vendor (Catalogue, City, State)	Dilution	
			WB	IHC/IF
β-Actin	Mouse	Affinity Biosciences (T0022, Cincinnati, USA)	1:1000	ND
HIF-1α	Rabbit	Abcam (ab2185, Cambridge, UK)	1:1000	ND
NRF2	Rabbit	Proteintech (16396-1-AP, Wuahan, China)	1:1000	1:200
KEAP1	Mouse	Proteintech (60027-1-Ig, Wuahan, China)	1:1000	ND
HO-1	Rabbit	Proteintech (10701-1-AP, Wuahan, China)	1:1000	1:200
NQO-1	Mouse	Proteintech (67240-1-Ig, Wuahan, China)	1:1000	ND
Lamin B	Rabbit	Proteintech (12987-1-AP, Wuhan, China)	1:1000	ND
MAP-2	Rabbit	Abcam (ab254264, Cambridge, UK)	1:1000	1:2000
MBP	Rabbit	Abcam (ab218011, Cambridge, UK)	1:1000	1:5000
IBA-1	Mouse	GeneTex (GTX632426, Texas, USA)	ND0	1:200
GFAP	Rabbit	Affinity Biosciences (AF6166, Cincinnati, USA)	ND	1:200
Cleaved Caspase-3	Rabbit	Cell Signaling Technology (9664, Danvers, MA)	1:1000	ND
BAX	Rabbit	Affinity Biosciences (AF0120, Cincinnati, USA)	1:1000	ND
BCL-2	Rabbit	Affinity Biosciences (AF6139, Cincinnati, USA)	1:1000	ND
HRP-Goat Anti-Mouse IgG		Proteintech (SA00001-1, Wuhan, China)	1:5000	ND
HRP-Goat Anti-Rabbit IgG		Proteintech (SA00001-2, Wuhan, China)	1:5000	1:200
Cy3-conjugated Goat Anti-Rabbit		Proteintech (SA00009-2, Wuhan, China)	ND	1:200
Cy3-conjugated Goat Anti-Mouse		Proteintech (SA00009-1, Wuhan, China)	ND	1:200
FITC-conjugated Goat Anti-Mouse		Proteintech (SA00003-1, Wuhan, China)	ND	1:200

ND = Not detected; WB = Western blot; IHC: Immunohistochemistry; IF: Immunofluorescence.
